# Increased externalizing and internalizing problems in children with sleep-disordered breathing

**DOI:** 10.1192/j.eurpsy.2021.1681

**Published:** 2021-08-13

**Authors:** E. Csábi, P. Benedek, V. Gaál

**Affiliations:** 1 Cognitive And Neuropsychology, Institute of Psychology, University of Szeged, Szeged, Hungary; 2 Sleep Lab, Heim Pál Children’s Hospital, Budapest, Hungary; 3 Department Of Paediatrics, University of Pécs, Pécs, Hungary

**Keywords:** sleep-disordered breathing, behavioral consequences, externalizing, internalizing behavioral problem

## Abstract

**Introduction:**

Sleep-disordered Breathing (SDB) is a spectrum disorder ranging from primary snoring to obstructive sleep apnea (OSA). One of the most common sleep-disorder in childhood, however remarkably little is known of the effect of SDB on behavioral functions.

**Objectives:**

The aim of our study to investigate the behavioral consequences of SDB compared to children with no history of sleep disorders.

**Methods:**

Two hundred thirty-four children aged 4-10 years participated in the study. The SDB group consists seventy-eight children, sixty-one of the them with OSA and seventeen with primary snoring (average age: 6,7 (SD = 1,83), 32 female/46 male), One hundred fifty-six children participated in the control group (average age: 6,57 years (SD = 1,46), 80 female/76 male). The two groups were matched by age and gender. We used the Attention Deficit Hyperactivity Disorder Rating Scale, Strength and Difficulty Questionnaire, and Child Behavior Checklist to assess the behavioral functions. Furthermore, the OSA-18 Questionnaire was administrated to support the diagnosis of SDB.

**Results:**

According to our results, children with SDB showed a significantly higher level of anxiety and depression and demonstrated significantly higher externalizing (such as attentional problems, hyperactivity, or social problems) and internalizing behavior problems (aggression, rule-breaking behavior).

**Conclusions:**

Due to the neurobehavioral consequences, our finding underlines the importance of the early diagnosis and treatment of sleep-disorder breathing.

**Disclosure:**

No significant relationships.

